# Overweight Is a Major Contributor to Atherosclerosis in Systemic Lupus Erythematosus Patients at Apparent Low Risk for Cardiovascular Disease: A Cross-Sectional Controlled Study

**DOI:** 10.1097/MD.0000000000002177

**Published:** 2015-12-07

**Authors:** Karim Sacre, Brigitte Escoubet, Maria-Christina Zennaro, Marie-Paule Chauveheid, Etienne Gayat, Thomas Papo

**Affiliations:** From the Département de Médecine Interne, Hôpital Bichat, Université Paris Diderot, PRES Sorbonne Paris Cité, Assistance Publique Hôpitaux de Paris, Paris, France (KS, M-PC, TP); INSERM U1149, Paris, France (KS, TP); Département Hospitalo-Universitaire FIRE (Fibrosis, Inflammation and Remodelling in Renal and Respiratory Diseases), Paris, France (KS, TP); Département de Physiologie, Hôpital Bichat, Université Paris Diderot, PRES Sorbonne Paris Cité, Assistance Publique Hôpitaux de Paris, INSERM U1138, Paris, France (BE); INSERM UMRS970, Paris Cardiovascular Research Center, Paris, France; Université Paris Descartes, Sorbonne Paris Cité, Paris, France; Assistance Publique-Hôpitaux de Paris, Hôpital Européen Georges Pompidou, Département de Génétique, Paris, France (M-CZ); and Département d’Anesthésie-Réanimation, Hôpitaux Universitaires Saint Louis—Lariboisière, 2 UMR-S 942, INSERM, 3 Université Paris Diderot, Paris, France (EG).

## Abstract

Cardiovascular disease (CVD) is the main cause of death in systemic lupus erythematosus (SLE) patients. We aimed to determine whether overweight (defined as a body mass index [BMI] > 25 kg/m^2^) contributed to subclinical atherosclerosis in SLE patients at low risk for CVD according to traditional factors.

Wall thickness of the internal carotid artery (ICWT) measured at the carotid bulb and carotid plaques were assessed in 49 SLE patients asymptomatic for CVD and 49 controls matched on Framingham score. Factors associated to ICWT were identified and multivariate analysis was performed.

SLE patients and controls displayed a low 10-year risk for CVD according to Framingham score (mean 1.9 ± 3.5 in SLE vs 1.8 ± 3.2% in controls, *P* = 0.37). ICWT (*P* < 0.001) and number of patients with carotid plaques (*P* = 0.015) were, however, higher in SLE patients as compared to controls. In multivariable analysis, SLE was an independent risk for a carotid atherosclerosis (OR [95% confidence interval, CI]: 3.53 [1.36–9.14]; *P* = 0.009). Older age, higher BMI, and higher Framingham score were associated with atherosclerosis in SLE patients in univariate analysis. In multivariate analysis, only the association with overweight remained significant (OR [95% CI]: 4.13 [1.02–16.75]; *P* = 0.047).

Overweight is a major contributor to atherosclerosis in SLE patients at apparent low risk for CVD.

## INTRODUCTION

In patients with systemic lupus erythematosus (SLE), cardiovascular disease (CVD) caused by atherosclerosis occurs more frequently and with earlier onset as compared to general population.^1–10^

Both traditional (such as age, hypertension, hypercholesterolaemia, or tobacco use) and SLE-related (such as disease duration or glucocorticoid use) factors have been identified as contributors to premature atherosclerosis in SLE.^3,11,12^

There is a strong relationship between obesity, defined by body mass index (BMI) > 30 kg/m^2^, and cardiovascular morbidity in general population.^13^ Interestingly, previous studies suggest that a high BMI is associated with atherosclerosis in SLE patients.^14–17^

In this study, we aimed to determine whether overweight (defined as a BMI > 25 kg/m^2^) contributed to subclinical atherosclerosis in SLE patients asymptomatic for CVD and at apparent low risk for CVD according to traditional factors.

## MATERIALS AND METHODS

### Study Participants

Forty-nine consecutive patients with SLE followed in the Department of Internal Medicine, Bichat Hospital, Paris-Diderot University, Paris were enrolled between February 2012 and February 2013. All subjects fulfilled at least 4 of the American College of Rheumatology criteria for SLE.^18^ Exclusion criteria consisted of known coronary disease or symptoms suggestive of CVD (angina, arrhythmia, congestive heart failure, stroke, and peripheral arterial disease).

Controls were healthy noncarrier relatives of pseudohypoaldosteronism type 1 patients from a clinical study that aimed to evaluate CVD with similar tools (PHACARV study, Assistance Publique-Hôpitaux de Paris, NCT00646828).^19^ All controls had undergone vascular ultrasound imaging between 2008 and 2011. None had coronary disease or symptoms suggestive of CVD.

The risk for cardiovascular events was calculated as the absolute risk within the next 10 years using the Framingham risk equation, which includes age, sex, total cholesterol level, high-density lipoprotein cholesterol level, smoking history, and systolic blood pressure. The first patient with SLE was considered with respect to Framingham score. The NCT00646828 clinical trial database was examined to find the first control patient of the same Framingham score. Matches were found for the 49 SLE participants.

Subjects were considered to have hypertension if they repeatedly had a systolic blood pressure of at least 140 mm Hg or a diastolic blood pressure of at least 90 mm Hg. Height and weight were measured, and the BMI was calculated as the weight in kilograms divided by the square of the height in meters. SLE disease activity was assessed using the safety of estrogens in lupus erythematosus national assessment-systemic lupus erythematosus disease activity index (SELENA-SLEDAI) score.^20^ The diagnosis of antiphospholipid syndrome was based on a history of venous and/or arterial thromboses or recurrent miscarriages in the presence of aPL antibodies in accordance with published criteria.^21^ Lupus nephritis diagnosis was based on International Society of Nephrology/Renal Pathology Society classification.^22^

The local ethics committee approved the study (Institutional Review Board [IRB 00006477] of HUPNVS, Paris 7 University, AP-HP). All patients provided written informed consent.

### Vascular Assessment

Vascular ultrasound study was performed in the context of care, in a temperature-controlled room after a 15-min rest (Vivid 7, General Electric, Horten, Norway). All subjects had fastened for at least 12 h before vascular evaluation. A single investigator conducted vascular measurements in controls and SLE patients. All data were analyzed offline (EchoPAC™, General Electric Ultrasound, Horten, Norway). Internal carotid (IC) artery was imaged in a longitudinal and cross-sectional view. Maximal thickness was measured as internal carotid wall thickness (ICWT) at the carotid bulb level at end diastole, as gated on ECG. Right and left values were averaged for each patient. Carotid plaques were defined as thickness >2 mm.

### Statistical Analysis

Continuous variables are expressed as mean and standard deviation (±SD). Categorical variables are expressed as frequencies and percentages. Data were compared between SLE patients and controls using Chi-squared test (or Fisher) for categorical variables and Student test (or Wilcoxon non-normally distributed) for continuous variables. ICWT was compared between SLE patients and controls using the Wilcoxon rank-sum test. Factors associated to ICWT were identified by use of multiple logistic regression (multivariate analysis). The Spearman rank correlation test was used to determine correlations between variables, with *r* being the Spearman correlation coefficient. Variables associated in univariate analysis with a *P*-value below 5% were considered for the multivariate model.

## RESULTS

### Atherosclerosis Assessed by Carotid Plaque Measurement Is High in SLE

Forty-nine SLE patients and 49 controls (CTL) were studied. The mean age of SLE subjects was 40.2 ± 10.6 years (35.4 ± 7.6 in controls, *P* = 0.023) and 41 (83.7%) were female (59.2% in controls, *P* = 0.013). Forty-eight patients (97.9%) received long-term glucocorticoids and 34 (69.4%) still used prednisone at a mean daily dose of 8.6 ± 3.3 mg (range: 5–17) that was stable for at least 3 months at time of study. Twenty-three (67.3%) had received immunosuppressive drugs at some point during follow-up. All SLE patients but 1 were receiving hydroxychloroquine.

Tobacco use, hypertension, cholesterol level, waist circumference, and BMI (calculated as the weight in kilograms divided by the square of the height in meters) were not statistically different between SLE patients and controls. Neither SLE patients nor controls had diabetes. The absolute risk of cardiovascular events occurring within the next 10 years according to the Framingham score was 1.9 ± 3.5% and 1.8 ± 3.2% in SLE patients and controls, respectively (*P* = 0.369). Only 1 SLE subject (2.0%) had a family history of CVD. In SLE patients, estimated glomerular rate filtration (eGRF) and HbA1c were, respectively, lower (86.6 ± 34.4 vs 98.7 ± 20.8 mL/min per 1.73 m^2^, *P* = 0.013) and higher (5.5 ± 0.5 vs 5.3 ± 0.4, *P* = 0.049) as compared to controls. Clinical characteristics of SLE patients and controls are shown in detail in Table 1.

**TABLE 1:**
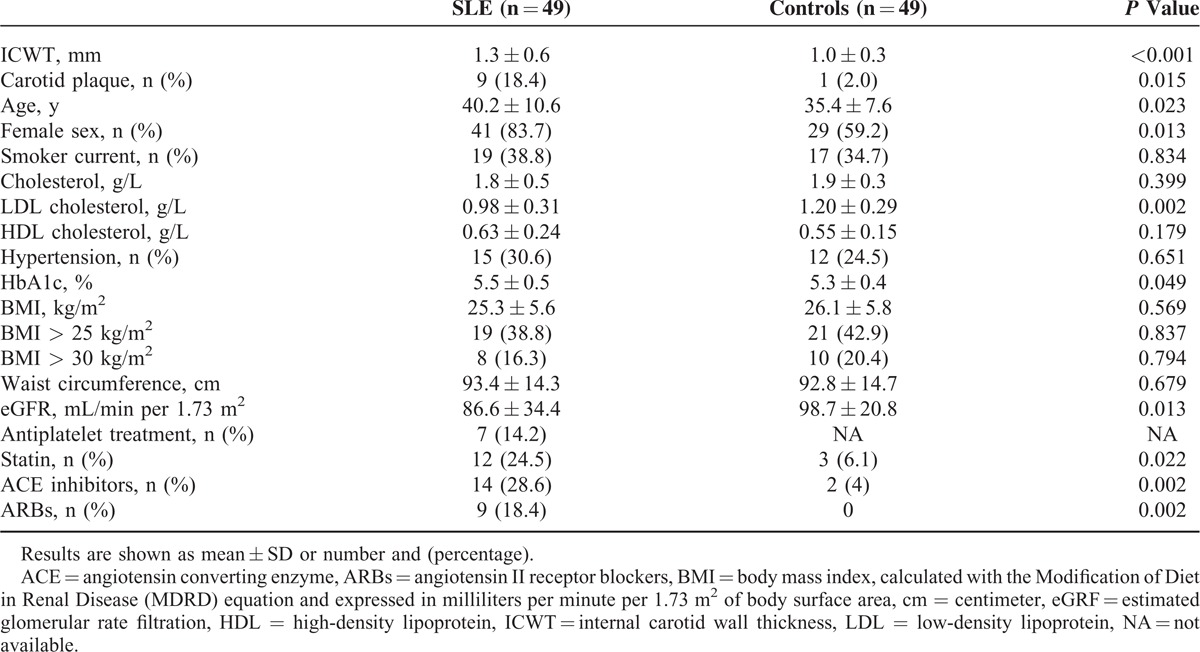
Characteristics of Systemic Lupus Erythematosus (SLE) Patients and Controls

ICWT was higher in SLE patients (1.3 ± 0.6 mm), as compared to controls (1 ± 0.3 mm, *P* < 0.001). Moreover, 9 (18.4%) SLE patients, but only 1 control (2.0%), displayed a carotid atherosclerotic plaque as defined as a local wall thickening >2 mm (*P* = 0.015). The multivariate analysis (Table 2) showed that SLE status was an independent risk factor for atherosclerosis by increasing of more than 3 times the risk for carotid plaques in patients (OR [95% confidence interval, CI]: 3.53 [1.36–9.14]; *P* = 0.009).

**TABLE 2:**
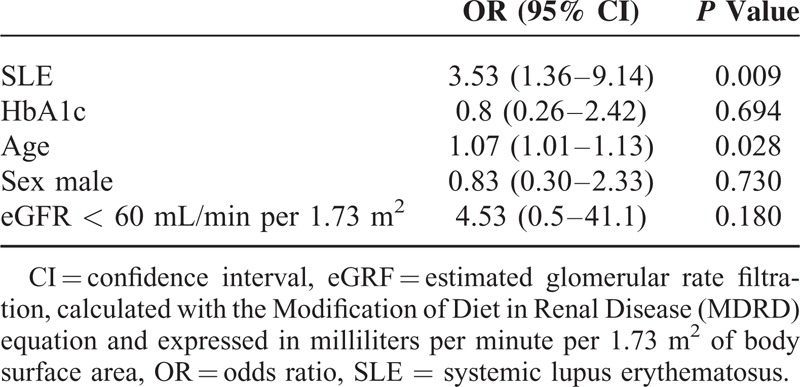
Multivariate Analysis of Risk Factors for Carotid Atherosclerosis

### Overweight Is an Independent Contributor to Atherosclerosis in SLE

**FIGURE 1:**
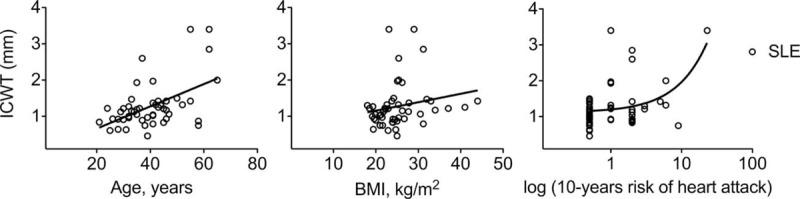
Carotid wall thickness correlates with age, BMI, and Framingham score in SLE. Correlation between ICWT and age (*r* = 0.42, 95% CI: 0.15–0.63, *P* = 0.003), BMI (*r* = 0.33, 95% CI: 0.05–0.57, *P* = 0.02), and 10-year risk of heart attack (*r* = 0.31, 95% CI: 0.03–0.55, *P* = 0.03) in SLE patients (white rounds). *r*, Spearman correlation coefficient. Ten-year risk of heart attack was calculated using the Framingham equation. BMI = body mass index, CI = confidence interval, ICWT = internal carotid wall thickness, SLE = systemic lupus erythematosus.

We separated SLE patients in 2 groups according of ICWT measurement: 1 group of SLE subjects with high ICWT (ICWT > median measurement; ie, >1.15 mm) and 1 group of SLE subjects with low ICWT (ICWT ≤ median measurement). In high ICWT group (n = 24), SLE patients were older (*P* = 0.012) and had a higher BMI (*P* = 0.042) and Framingham score (*P* = 0.043) as compared to patients with low ICWT (n = 25). No statistical difference between groups was observed regarding factors more directly associated with SLE disease such as duration of disease, SLE disease activity assessed using the SELENA-SLEDAI score, eGFR, proteinuria, antiphospholipid status, or steroid treatment (Table 3). Moreover, ICWT significantly correlated with age (*r* = 0.42, 95% CI: 0.15–0.63, *P* = 0.003), BMI (*r* = 0.33, 95% CI: 0.05–0.57, *P* = 0.019), and Framingham score (*r* = 0.31, 95% CI: 0.03–0.55, *P* = 0.027; Fig. 1). In the multivariate analysis (Table 4), only overweight (ie, BMI > 25 kg/m^2^) was associated with a high ICWT status (OR [95% CI]: 4.13 [1.02–16.75]; *P* = 0.047) in SLE. Using multivariate linear regression, BMI > 25 kg/m^2^ was independently associated with ICWT after adjustment for age, and Framingham score (mean difference: 1.42 [0.35–5.77], *P* = 0.029). Overall, the risk of having carotid atherosclerosis increased of 16% for each kg/m^2^ of BMI taken in SLE patients (OR [95% CI]: 1.16 [1.01–1.33]).

**TABLE 3:**
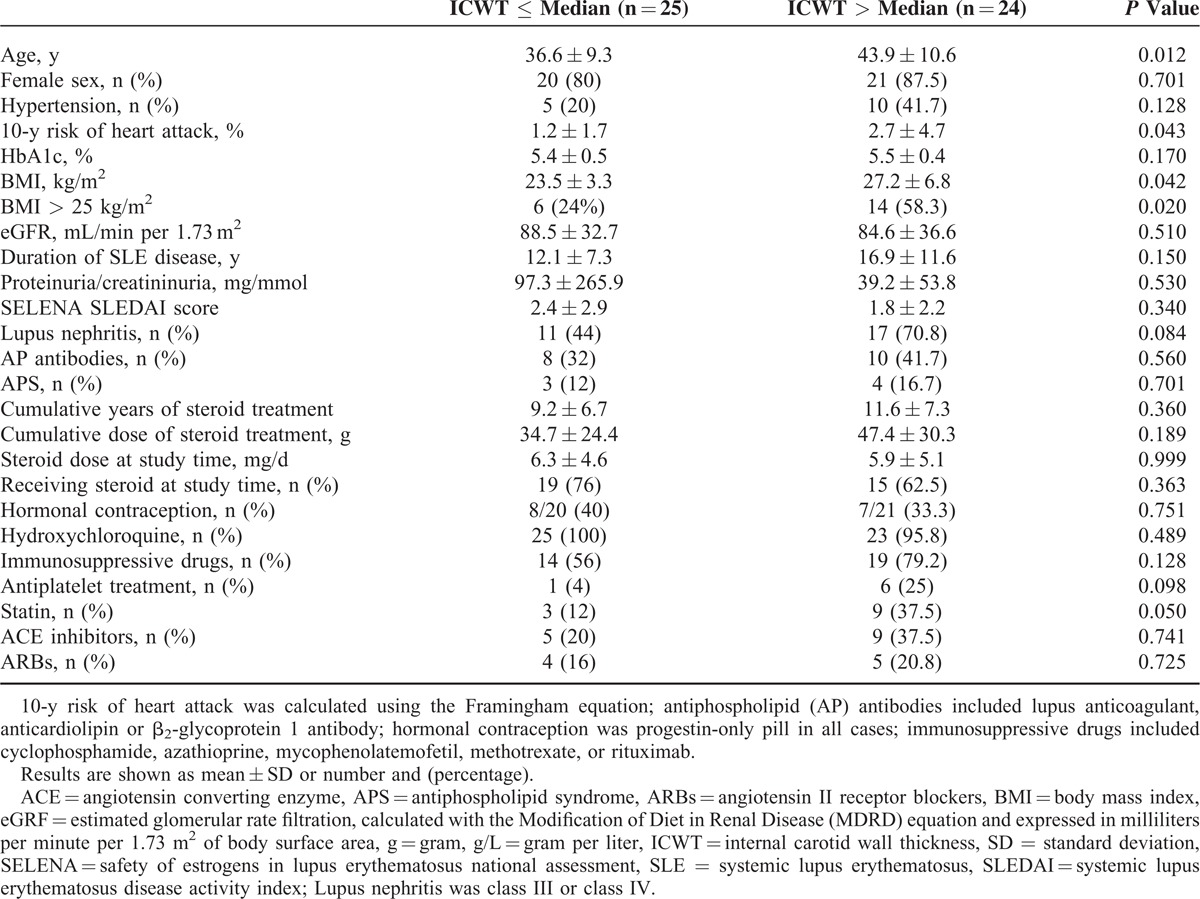
Factors Associated With Carotid Atherosclerosis in SLE Patients at Low Risk for Cardiovascular Disease

**TABLE 4:**
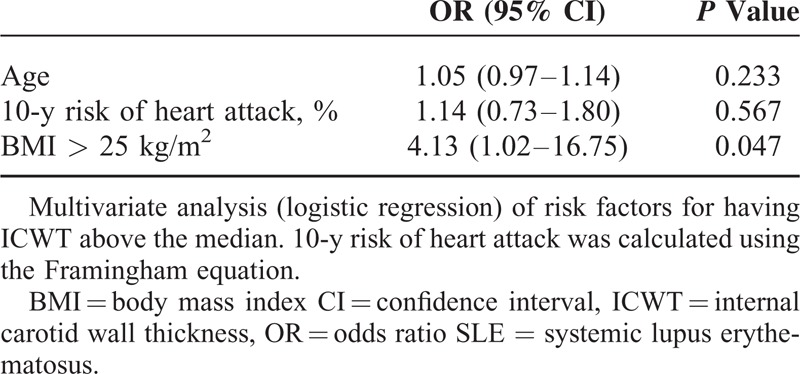
Multivariate Analysis of Risk Factors for Carotid Atherosclerosis in SLE Patients

## DISCUSSION

Our study shows that overweight is a major contributor to atherosclerosis in SLE patients at low calculated risk for CVD according to traditional factors. The association of a higher BMI with atherosclerosis has been recognized previously in both general population and SLE patients. Our data reinforce the notion that body weight should taken into account in all patients with SLE to prevent the occurrence of CVD.

ICWT is the direct measurement of maximal arterial wall thickness at the IC artery and is the reference method to identify IC atheroma. In our study, carotid wall thickness was measured at the carotid bulb and, for giving unequivocal evidence of atherosclerosis; carotid plaque was defined as an ICWT >2 mm (instead of 1.5 mm as usually accepted).^23^

ICWT differs from carotid intima media thickness (IMT). Carotid IMT refers to the semi-automatic measurement of wall thickness usually obtained from the posterior wall of common carotid artery (CCA). IMT has been used as a surrogate marker of atherosclerosis while ICWT gives unequivocal evidence of atherosclerosis (ie, plaque).^24^ Moreover, carotid plaques at IC appeared to represent a stronger marker of coronary vessel disease than IMT. Indeed, a meta-analysis of 11 population-based studies including 54,336 patients showed that carotid plaque, when compared with IMT (inclusive of CCA, bulb, and/or IC depending on the study), had a significantly higher diagnostic accuracy for the prediction of future myocardial infarction.^25^

The majority of studies interested in atherosclerosis associated with autoimmune diseases limited ultrasound examination to the CCA for IMT measurement. However, all large studies in SLE patients found a lower or normal IMT but a significantly higher rate of carotid atherosclerotic plaques compared with age-matched and sex-matched controls.^3,26,27^ Moreover, a recent study showed that carotid atherosclerotic lesions can be found frequently in absence of increased IMT in SLE.^28^ Accordingly in our study, mean IMT was normal in SLE patients (0.53 ± 0.11 mm) and did not differ statistically from controls (0.51 ± 0.07 mm, *P* = 0.29) while carotid wall thickness was higher in SLE patients (*P* = 0.0007) and 9 SLE patients (but only 1 control), had a carotid plaque (*P* = 0.015).

Overabundance of fat tissue may create unhealthy levels of hormones, proteins, and cytokines that may not only elevate the risk of CVD but also promote other disease processes. Indeed, obesity appears to be a major environmental factor contributing to the onset and progression of autoimmune diseases^29^ and is independently associated with inflammation markers in lupus patients.^30^ Adipose tissue secretes cytokines such as TNF-α, IL-6, and IL-10^31^ known to be involved in both SLE^32^ and atherosclerosis.^33^ Furthermore, BMI has been associated to increased serum level of dysfunctional pro-inflammatory HDL and free fatty acids, and to insulin resistance in patients with SLE,^34–36^ all known to contribute to atherosclerosis.

Metabolic syndrome is highly prevalent in SLE, even in young patients recently diagnosed,^37^ and appeared to contribute to increased cardiovascular risk in SLE.^38^ Although our study does not provide indication on fat distribution, SLE patients with overweight had an elevated waist circumference (103.8 ± 15.4 vs 86 ± 7.1 cm as compared to SLE patients without overweight, *P* < 0.0001).

Although corticosteroid treatment is well known to be associated with weight gain, we failed to identify glucocorticoid as an independent contributor to overweight in our study. Other studies showed only a limited or no association between corticosteroids and the presence of the metabolic syndrome or insulin resistance in patients with SLE.^39,40^ Eventually, a better control of inflammation may help to counterbalance the deleterious effect of corticosteroids on weight gain.

Our study has limitations. It included a relatively small number of patients with SLE, so larger studies may yield different results. In addition, longitudinal studies are clearly needed to better define the importance of overweight regarding CVD risk in SLE patients in addition to known risk factors. Eventually, despite the matching on Framingham score, the difference concerning age and sex between SLE and control group should be underlined.

In conclusion, overweight is a major contributor to atherosclerosis in SLE patients at apparent low risk for CVD according to traditional risk factors. Identifying SLE patients at a higher risk for CVD is mandatory. Body weight control should be a specific target in clinical practice in order to prevent the occurrence of atherosclerosis and its deleterious consequences in all patients with SLE.
